# Supplementary material for: Prevalence of anemia among reproductive women in different social group in India: Cross-sectional study using nationally representative data
Source: PLoS One. 2023 Feb 2;18(2):e0281015. doi: 10.1371/journal.pone.0281015 (PMC9894404; doi:10.1371/journal.pone.0281015)
Supplement: Attachment — Submitted filename: Answer to Editor 2.docx [file pone.0281015.s001.docx]

We've checked your submission and before we can proceed, we need you to address the following issues:

1. It appears that your ORCiD iD has not been validated in your Editorial Manager account and we are unable to proceed until that step is complete.

To validate your ORCiD iD in Editorial Manager, please follow the steps below:

1. In your Editorial Manager account, please go to ‘Update my Information’ (in the upper left-hand corner of the main menu), and click on the Fetch/Validate link next to the ORCiD field.

2. This link will take you to the ORCiD site and allow you to create a new iD or authenticate a pre-existing iD in Editorial Manager.

For additional instructions, please watch the following video for a step-by-step demonstration: https://www.youtube.com/watch?v=_xcclfuvtxQ

Answer: Added
2. Please amend the title either on the online submission form or in your so that they are identical.
Answer: Corrected

3. We note that you have uploaded a new version of figure 1 in your manuscript, "Figure_Change.docx"
Answer: Yes
At this time, we request that you clarify what program or programs, if any, were used to create the figures which you have mentioned were created for this publication, and what the source or sources of the shapefiles and basemaps used to create them were.
Answer: Base map collect from the open sources of Survey of India [www.surveyofindia.gov.in](http://www.surveyofindia.gov.in)
We look forward to your reply.


4. We note your Data Availability Statement reads: "OPEN SOURCES DATA, WHIS IS AVAILABLE FOR ALL PEOPLE."
Answer: Yes, This open sources data has been taken from <http://rchiips.org/nfhs/>

<http://rchiips.org/nfhs/nfhs4.shtml>

<http://rchiips.org/nfhs/NFHS-5Reports/NFHS-5_INDIA_REPORT.pdf>

Before we can proceed, please provide us with the relevant URLs, DOIs, or accession numbers necessary to access your data set.
